# Synthesis and Detection of Oxygen-18 Labeled Phosphate

**DOI:** 10.1371/journal.pone.0018420

**Published:** 2011-04-04

**Authors:** Eric S. Melby, Douglas J. Soldat, Phillip Barak

**Affiliations:** Department of Soil Science, University of Wisconsin-Madison, Madison, Wisconsin, United States of America; University of Pennsylvania, United States of America

## Abstract

Phosphorus (P) has only one stable isotope and therefore tracking P dynamics in ecosystems and inferring sources of P loading to water bodies have been difficult. Researchers have recently employed the natural abundance of the ratio of ^18^O/^16^O of phosphate to elucidate P dynamics. In addition, phosphate highly enriched in oxygen-18 also has potential to be an effective tool for tracking specific sources of P in the environment, but has so far been used sparingly, possibly due to unavailability of oxygen-18 labeled phosphate (OLP) and uncertainty in synthesis and detection. One objective of this research was to develop a simple procedure to synthesize highly enriched OLP. Synthesized OLP is made up of a collection of species that contain between zero and four oxygen-18 atoms and, as a result, the second objective of this research was to develop a method to detect and quantify each OLP species. OLP was synthesized by reacting either PCl_5_ or POCl_3_ with water enriched with 97 atom % oxygen-18 in ambient atmosphere under a fume hood. Unlike previous reports, we observed no loss of oxygen-18 enrichment during synthesis. Electrospray ionization mass spectrometertry (ESI-MS) was used to detect and quantify each species present in OLP. OLP synthesized from POCl_3_ contained 1.2% P^18^O^16^O_3_, 18.2% P^18^O_2_^16^O_2_, 67.7% P^18^O_3_^16^O, and 12.9% P^18^O_4_, and OLP synthesized from PCl_5_ contained 0.7% P^16^O_4_, 9.3% P^18^O_3_^16^O, and 90.0% P^18^O_4_. We found that OLP can be synthesized using a simple procedure in ambient atmosphere without the loss of oxygen-18 enrichment and ESI-MS is an effective tool to detect and quantify OLP that sheds light on the dynamics of synthesis in ways that standard detection methods cannot.

## Introduction

The lack of multiple stable isotopes of P has led researchers to study the ratio of ^16^O and ^18^O in phosphate, the dominant form of P in the natural environment. The naturally occurring ratio of ^18^O/^16^O bonded to phosphorus in phosphate, expressed as δ^18^O_p_, has been used to infer the source of P in water bodies [1]–[6]. Gruau et al. [7] found substantial variability of δ^18^O_p_ within P sources and considerable overlap of δ^18^O_p_ among different P sources and therefore questioned the value of this method as a research tool. Based on the variability and overlap of δ^18^O_p_ in natural sources, some researchers have used a method that enriches phosphate in oxygen-18, making it very distinct from any other P sources. The synthesis of oxygen-18 labeled phosphate (OLP) is generally carried out by hydrolyzing either PCl_5_ or POCl_3_ with water enriched with oxygen-18. This reaction is typically carried out in an atmosphere devoid of moisture to prevent the highly reactive PCl_5_ or POCl_3_ from reacting with atmospheric water [8]–[11]. Middleboe and Saaby Johansen [12] reported that up to one-half of the ^18^O enrichment was sacrificed when POCl_3_ was hydrolyzed with H_2_^18^O in ambient atmosphere.

Analysis of the ^18^O/^16^O ratio of OLP has been previously carried out through the use of optical emission spectroscopy [9], [13], [14], in which the OLP present in solution, plant tissue (after being extracted with 0.5 M HCl), or soil (after being extracted with anion exchange resin) is precipitated as struvite (MgNH_4_PO_4_·6H_2_O) [9], [14]. This precipitate is pyrolyzed to carbon monoxide, which is analyzed for its ^18^O/^16^O ratio by optical emission spectroscopy [14]. Synthesized OLP is made up of a collection of species that contain between zero to four oxygen-18 atoms. Optical emission spectroscopy is only capable of determining an overall ^18^O/^16^O ratio and is not able to distinguish between the various OLP species. The use of electrospray ionization mass spectrometry (ESI-MS) has also been presented as a viable means to determine the ^18^O/^16^O ratio of OLP and as a detection tool that can differentiate between OLP species. OLP dissolved in water was analyzed by ESI-MS and the results were compared to those obtained with ^31^P NMR analysis. The authors reported good agreement between the ESI-MS and ^31^P NMR results [11]. However, ESI-MS is sensitive at mg L^−1^ P levels, which is lower than ^31^P NMR limits of detection.

The first objective of this research was to develop a simpler, efficient technique for the synthesis of OLP. The second objective was to use ESI-MS to quantify the individual OLP species present in synthesized OLP compounds. A final objective included determining the stability of OLP stored in sterile conditions over a period of several months.

## Methods

The synthesis of OLP was carried out after consultation with the University of Wisconsin-Madison Environment, Health and Safety Department (Madison, Wisconsin) as POCl_3_ and PCl_5_ are both highly toxic chemicals. A chemical resistant suit, chemical resistant gloves, and a positive pressure, self-contained breathing apparatus (Scott Health and Safety, Monroe, North Carolina) were worn during the handling of these chemicals and during their reaction with water to prevent any contact or inhalation of fumes.

### OLP Synthesis from POCl_3_

The synthesis of OLP from POCl_3_ was carried out in a 25×200 mm glass test tube contained within a 250 mL Erlenmeyer flask filled with 150 mL of water to moderate the temperature. The flask and test tube were placed on a combination stir/hot plate within a fume hood. OLP was synthesized through the following spontaneous and highly exothermic reaction:
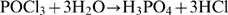



2.00 g of water with 97 atom % ^18^O (Sigma-Aldrich, St. Louis, Missouri) were added to the test tube, followed by 4.87 g of POCl_3_ (Pfaltz and Bauer Inc., Waterbury, Connecticut) added drop wise while stirring with a magnetic stir bar. Labeled water was added in 10% excess to ensure that the POCl_3_ reacted completely with water containing ^18^O. After the vigorous reaction subsided, the test tube was heated for 2 h at 100°C with stirring to remove HCl produced during the reaction. Following heating, the test tube was allowed to cool to room temperature and 5 M KOH was added to raise the pH to 4.7. The KH_2_PO_4_ product was diluted with ultrapure water, and the P concentration of this solution was determined using an IRIS Advantage inductively coupled plasma optical emission spectrometer (ICP-OES) (Thermo Jarrell Ash, Franklin, Massachusetts). The reaction was carried out with pure POCl_3_, and with POCl_3_ stored for a period of 6 and 17 months after it was initially opened to determine if there was any effect of long term POCl_3_ storage on the OLP species composition.

### OLP Synthesis from PCl_5_

The synthesis of OLP from PCl_5_ was carried out using the same procedure as described above, but this time reacting 2.50 g of PCl_5_ (Sigma-Aldrich, St. Louis, Missouri) with 1.00 g of water containing 97 atom % ^18^O (Sigma-Aldrich, St. Louis, Missouri) through the following spontaneous and highly exothermic reaction:
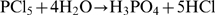



The reaction was carried out with freshly opened PCl_5_, and PCl_5_ that was stored for a period of 17 months after it was initially opened.

### Quantification of Species Using ESI-MS and Long-Term Stability of OLP in Sterile Water

**Figure 1:**
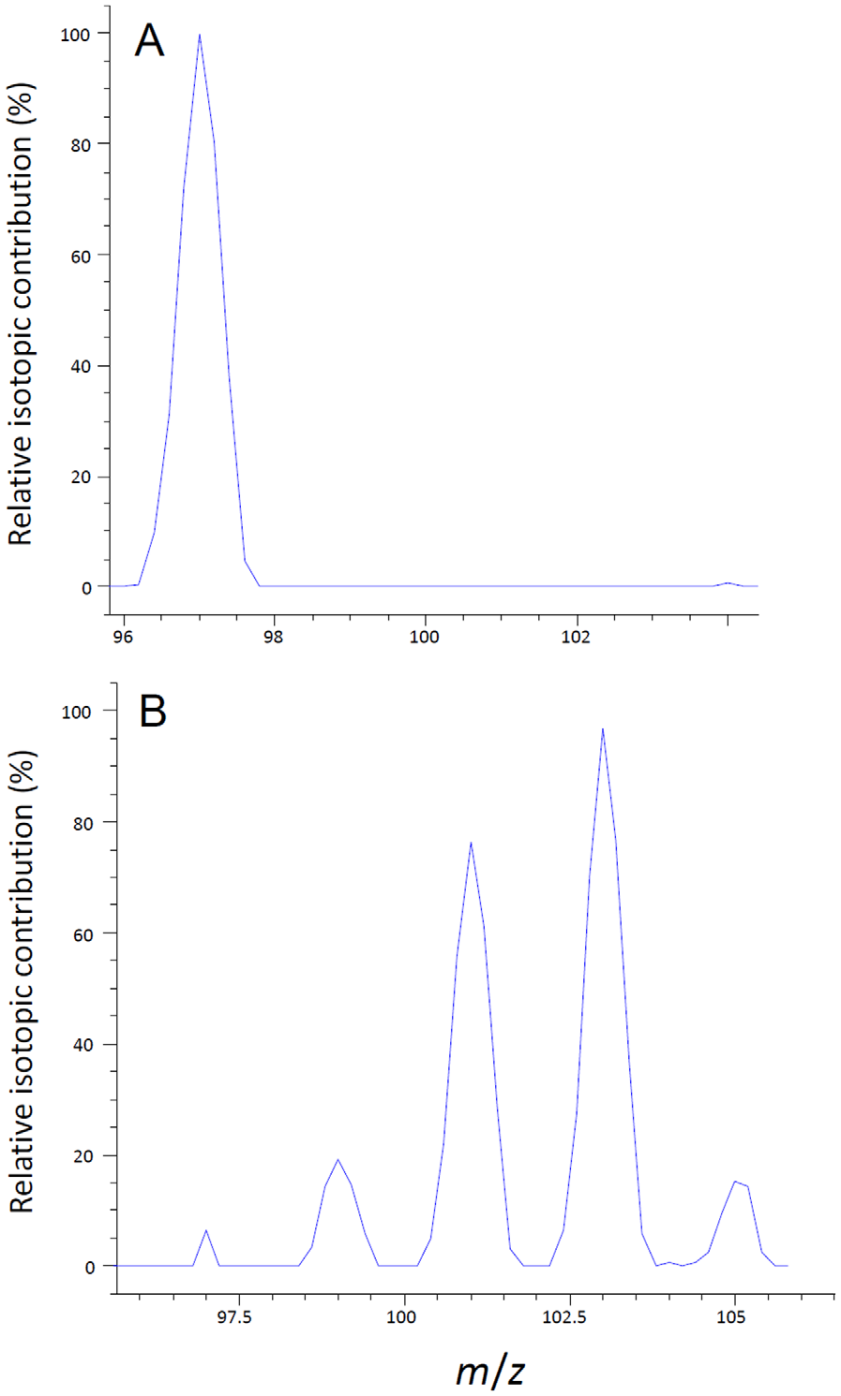
Electrospray ionization spectra of labeled and unlabeled phosphate species. Relative abundance of (a) unlabeled phosphate (*m/*z = 97) and (b) ^18^O-labeled phosphate species (*m/z* = 99, 101, 103, and 105) as determined by ESI-MS.

Electrospray ionization mass spectrometry (ESI-MS) was used to establish a ratio of each of the species present in the synthesized OLP. All samples were analyzed using an Agilent 1100 LC-MSD SL single-quadrupole model 1946D mass spectrometer (Agilent Technologies, Santa Clara, California). Table 1 lists the liquid chromatography (LC) and mass spectrometer (MS) conditions used for this analysis. The samples were diluted to approximately 60 mg L^−1^ P prior to analysis and analyzed in duplicate injections. Oxygen labeled phosphate eluted from the LC beginning at ∼4.2 min and ending at 4.5 min after injection. During OLP analysis by ESI-MS, the *m/z* range monitored was 90–120, as the peaks of interest were *m/z* 97 (H_2_P^16^O_4_^−^), 99 (H_2_P^16^O_3_^18^O^−^), 101 (H_2_P^16^O_2_^18^O_2_^−^), 103 (H_2_P^16^O^18^O_3_^−^), and 105 (H_2_P^18^O_4_^−^). All other peaks within this range were negligible (Fig. 1). The area of each spectra for individual phosphate species were compared to the cumulative area of all the phosphate species allowing for the calculation of the relative contribution of each phosphate species shown in Figure 1. This relative information was used with the total P concentration of the sample to calculate the concentration of phosphate species in solution.

**Table 1:** Liquid chromatography and mass spectrometry conditions used for electrospray ionization mass spectrometer analysis of oxygen-18 labeled phosphate.

Parameter	Condition
*Liquid chromatography*	
Column	Waters IC-Pak Anion HR, 4.6×75 mm, 6 µm particle size (Waters Corp., Milford, Massachusetts)
Mobile phase	Isocratic: 25% acetonitrile, 75% 50 mM ammonium bicarbonate (pH 10)
Flow rate	0.5 mL min^−1^
Column temperature	30°C
*Mass spectrometry*	
Injection volume	15 µL
Source voltage	2250 V
Drying gas	Nitrogen, 350°C, 40 L min^−1^, 40 psi
Ionization	Electrospray
MS detection	Single ion recording (SIR) mode

To monitor the stability of OLP solutions in a sterile environment, aqueous OLP solutions were diluted with autoclaved ultrapure water, placed in autoclaved polypropylene containers, and stored in darkness at 4°C.

## Results

Data from the ESI-MS analysis of OLP synthesized from POCl_3_ and PCl_5_ are shown in Tables 2 and 3, respectively, along with the theoretical synthesis values. Theoretical values were calculated assuming completely random arrangement of the oxygen atoms from labeled water and assuming no P-O bonds of POCl_3_ were broken. We observed almost no loss of oxygen-18 enrichment when synthesizing OLP from pure POCl_3_ (Table 2). However, OLP synthesized from POCl_3_ stored six months had an oxygen-18 content of 62.2% and POCl_3_ stored 17 months had an oxygen-18 content of 55.3%. This enrichment loss can probably be attributed to water vapor entering the POCl_3_ container either when the bottle was opened or throughout the storage period. There was no loss of oxygen-18 enrichment when synthesizing OLP from PCl_5_ that had been stored for 17 months after it was initially opened (Table 3).

**Table 2:** Amount of each oxygen labeled phosphate (OLP) species present in OLP synthesized with pure POCl_3_ during two separate synthesis events with either fresh POCl_3_ or with POCl_3_ previously opened and stored containers as compared to the theoretical values.

OLP species	Amount of species present in OLP				
	Theoretical†	June 2009		May 2010	
		Fresh	Stored‡	Fresh	Stored§
*–––– m/z ––––*	*––––––––––––––––––––––––* % *––––––––––––––––––––––––*				
97	0.0	0.0	1.0	0.0	21.7
99	0.3	1.2	8.7	3.7	4.0
101	8.5	18.2	36.3	23.6	19.36
103	91.1	67.7	48.5	52.6	51.6
105	0.2	13.0	5.58	20.2	13.4
Total ^18^O %	72.8	73.1	62.2	72.3	55.3

†Calculated assuming no P-O bonds in POCl_3_ were broken during the reaction.

‡Opened and stored for 6 months.

§Opened and stored for 17 months.

**Table 3:** Amount of each oxygen labeled phosphate (OLP) species present in OLP synthesized from PCl_5_ during two separate synthesis events (December 2008 and May 2010) as compared to theoretical values.

OLP species	Amount of species present in OLP		
	Theoretical	December 2008	May 2010†
*–––– m/z ––––*	*–––––––––––––––––––––––* % *––––––––––––––––––––––––*		
97	0.0	0.7	0.0
99	0.0	0.0	0.0
101	0.5	0.0	0.0
103	11.0	9.3	12.2
105	88.5	90.0	87.8
Total ^18^O %	97.0	97.0	97.0

† The May 2010 synthesis event used PCl_5_ which was opened and stored for 17 months, while the PCl_5_ used in the December 2008 synthesis was opened immediately prior to synthesis.

Based on the results from the reaction of PCl_5_ with 97 atom % ^18^O water carried out in ambient atmosphere (Table 3) in December 2008, completely labeled P^18^O_4_ made up 90.0% of the species present and the overall oxygen-18 content of the OLP was 97.0%.

There was very little deviation between the theoretical values and actual values of the OLP species present in OLP synthesized from PCl_5_ (Table 3). However, large differences between the theoretical values and actual values of the amount of each OLP species present in OLP synthesized from POCl_3_ were observed (Table 2). One unexpected result of the synthesis of OLP from POCl_3_ was the formation of a significant amount of P^18^O_4_ (*m/z* 105), which made up 13.0% of the OLP synthesized from fresh POCl_3_. This is noteworthy because the POCl_3_ starting material contains one oxygen atom that was expected to remain bonded to the P atom, preventing the formation of P^18^O_4_. The natural abundance of oxygen-18 at 0.2% does not explain this level of P^18^O_4_ production. After observing these results, we hypothesized that the amount of energy released during the reaction between POCl_3_ and water was enough to break the P-O bond in POCl_3_, thus resulting in a random assortment of the oxygen-16 and oxygen-18 atoms bonded to the P atoms. We predicted the expected synthesis outcome based on all the P-O bonds in POCl_3_ breaking and the total amount of oxygen-18 (73.0%) and oxygen-16 (27.0%) contained in the synthesized OLP, (Table 4). Based on the differences between the expected and actual results, especially for the OLP species at *m/z* 103 (P^18^O_3_^16^O) and 105 (P^18^O_4_), it appears that some of the O atoms present in the POCl_3_ starting material remained bonded to the P atom during the reaction with water, while the bond between other POCl_3_ O atoms and P was broken.

**Table 4:** Calculation of the expected amount of each oxygen labeled phosphate (OLP) species formed with completely random assortment of the oxygen atoms and comparison to the actual results from OLP synthesized with fresh POCl_3_ in December 2008.

OLP species	^16^O	^18^O	Combinations†	Probability‡	Expected composition§	Observed composition
*m/z*	# of atoms				*––––––––––––––* % *––––––––––––––*	
97	4	0	1	0.00531	0.53	0.00
99	**3**	1	4	0.01437	5.75	1.18
101	2	2	6	0.03885	23.31	18.18
103	1	3	4	0.10504	42.01	67.69
105	0	4	1	0.28398	28.39	12.95

†Number of different ways this combination of oxygen-16 and oxygen-18 atoms can be arranged around the phosphorus atom.

‡Probability was calculated by taking the amount of each oxygen atom (62.2 and 37.8% for oxygen-18 and oxygen-16, respectively) to the power of the number of that atom contained in the OLP species (e.g., the probability for *m/z* 99 is 0.378^3^ * 0.622^1^ = 0.033594).

§Calculated by multiplying the number of combinations by the probability.

The extent of broken P-O bonds in POCl_3_ during OLP synthesis was calculated using an implicit equation that reduced the overall 97% oxygen-18 enrichment by an amount equal to the extent of broken bonds supplying additional oxygen-16. This calculation allowed those POCl_3_ molecules with broken P-O bonds to react with four O atoms from the O isotope pool and those with no broken P-O bonds to react with three O atoms. Solutions to the equation were iterated comparing the measured isotopic ratios with those predicted until the sum of squares of the differences was minimized. The results show close agreement between the actual amount of each OLP species synthesized and the predicted values (Table 5). The results of the calculation suggest that 17.9% of the P-O bonds in POCl_3_ were broken during the December 2008 synthesis, while 38.4% of the bonds were broken during the May 2010 synthesis.

**Table 5:** Comparison between synthesis results and theoretical values of P-O bonds in POCl_3_ broken during the synthesis of oxygen-18 labeled phosphate (OLP) using POCl_3_.

OLP species	December 2008		May 2010	
	Theoretical† Actual Theoretical‡ Actual
*–––– m/z ––––*	*––––––––––––––––––––––––––––––––––––* % *––––––––––––––––––––––––––––––––––*			
97	0.1	0.0	0.2	0.0
99	1.7	1.2	3.5	3.7
101	18.1	18.2	22.4	23.6
103	67.5	67.7	52.8	52.6
105	12.7	13.0	21.1	20.2

†Calculated assuming 17.9% of the POCl_3_ P-O bonds were broken.

‡Calculated assuming 38.4% of the POCl_3_ P-O bonds were broken.

The OLP solutions were synthesized with fresh POCl_3_ and PCl_5_ in December 2008 and they were analyzed by ESI-MS 16 months later in April 2010. After the 16 month period, the total oxygen-18 content of the OLP solutions synthesized from POCl_3_ and PCl_5_ were 73.0% and 97.0%, respectively. This indicates that there was no O exchange between phosphate and water, which is in agreement with Blake et al. [15] and McLaughlin and Paytan [16].

## Discussion

These synthesis reactions were carried out in ambient air under a fume hood using a fairly simple procedure as compared to previous synthesis attempts. Middleboe and Saaby Johansen [12] reported a loss of about one-half of the expected oxygen-18 enrichment when synthesizing OLP in ambient atmosphere from POCl_3_, which they suggested should be expected for any OLP synthesis carried out in ambient atmosphere. Based on the reaction of POCl_3_ with water that contains 97 atom % ^18^O, it would be expected that the maximum amount of oxygen atoms being oxygen-18 would be 72.8% (assuming that 0.2% of the POCl_3_ oxygen atoms are oxygen-18 based on the natural abundance of oxygen-18). Additionally, based on the reaction between PCl_5_ and 97 atom % ^18^O water, one would expect the maximum amount of oxygen-18 atoms to be 97%. Following Middleboe and Saaby Johansen [12], we would have expected only 36.5% of the phosphate oxygen atoms in the OLP synthesized from POCl_3_ and 48.5% of the phosphate oxygen atoms in the OLP synthesized from PCl_5_ would be oxygen-18. However, our enrichment levels were substantially greater for both POCl_3_ and PCl_5_ (Table 2). In fact, we observed almost no loss of oxygen-18 enrichment by performing the reactions in ambient air. It is possible that the loss of oxygen-18 enrichment reported by Middleboe and Saaby Johansen [12] when synthesizing OLP in ambient atmosphere was the result of decreased POCl_3_ purity as the result of reaction with atmospheric water prior to synthesis, as we noted after running the reactions with POCl_3_ that had been opened and stored for several months.

Ray [10] carried out a reaction of water and PCl_5_ in dioxane and took extreme care to exclude atmospheric water from the reaction; he reported a yield of 85±5% P^18^O_4_. The results obtained using the current procedure resulted in a yield of P^18^O_4_ that was equal to or greater than that reported using the more difficult procedure of Ray [10] (Table 3). Similarly, Risley and Van Etten [8] also carried out this reaction, this time excluding atmospheric water using drying tubes, and obtained an overall oxygen-18 enrichment of 90%. This enrichment is 9% less than what would be expected from the reaction as 99 atom % ^18^O water was used. Again, the simpler procedure detailed in this study resulted in an improved oxygen-18 enrichment of 97.0%, which represents no loss of enrichment as 97 atom % ^18^O water was used in this study.

Natural abundance methods rely on a δ^18^O_p_ range of about 10‰ to 25‰ between different phosphate sources, which represents a narrow range. Additionally, the δ^18^O_p_ ranges of many phosphate sources overlap one another, making it very difficult to infer sources of phosphate. The OLP synthesized from fresh POCl_3_ and PCl_5_, on the other hand, had a δ^18^O_p_ value around 1,350,000‰ and 16,120,000‰, respectively. This huge separation from other potential phosphate sources should make it possible to differentiate between OLP and other phosphate sources.

Our use of ESI-MS proved to be an effective means to quantify the products of OLP synthesis. ESI-MS analysis provides information about the individual OLP species composition, which sheds light on reaction dynamics that would not be known by simply analyzing an overall O^18^/O^16^ ratio. For example, we determined that although the total ^18^O composition of our synthesized compounds matched the predicted amount, the ^18^O composition of the individual species differed, indicating that the assumptions of either complete, or no P-O bond-breakage in POCl_3_ were not met. Bond breakage ranged from 18 to 38%, possibly due to different reaction temperatures during the very violent and exothermic reaction caused by drop wise additions of labeled water.

There have been several previous reports on the stability or decay of OLP in an aqueous solution. Results have ranged from the exchange of oxygen between water and phosphate reaching equilibrium in 3 h [17] to reports of a much slower half-life period of 100 months [12]. The latter result is in closer agreement with more recent publications that state oxygen exchange between phosphate and water under standard environmental conditions does not occur in the absence of biological mediation [15], [16]. Our results support the findings of the later studies.

The results from the reactions carried out in ambient atmospheric conditions between POCl_3_ or PCl_5_ and 97 atom % ^18^O water show that over 99% of the OLP molecules contained at least one oxygen-18 atom, and would therefore be potentially useful for creating and tracing compounds in the laboratory or possibly the environment. Overall, the results indicate that OLP highly enriched in oxygen-18 can be synthesized under ambient atmosphere using a simple procedure and this product does not lose its label over time in the absence of microbial mediation. ESI-MS can be used to easily and effectively gain information about the individual OLP species composition.
